# A specialized training program enhances university students’ knowledge, attitudes, and practices on volunteering: evidence from Oman

**DOI:** 10.3389/fsoc.2025.1636370

**Published:** 2025-08-20

**Authors:** Emad Farouk Saleh, Wafa Said Al-Maamari, Ahmed Thabet Helal Ibrahim, Magdy M. Mostafa, P. Padma Sri Lekha, E. P. Abdul Azeez

**Affiliations:** ^1^College of Arts and Social Sciences, Sultan Qaboos University, Seeb, Oman; ^2^School of Social Sciences and Languages, Vellore Institute of Technology, Vellore, India

**Keywords:** volunteering, awareness, Oman, knowledge, attitude and practice, training program, university students

## Abstract

The decreasing involvement of youth in voluntary practices necessitates training and mobilizing them for voluntary activities. Therefore, the present study aimed to develop and test the efficacy of a culture-sensitive training program on enhancing knowledge, attitude, and practice (KAP) of volunteering among university students in Oman. We adopted a quasi-experimental design with a pre-post-test on the KAP of volunteering and designed a specialized training program spanning 10 weeks. Training was given to two groups of university students independently during different semesters to test the efficacy of the program (group 1 and group 2). The results of the paired *t*-test suggested a significant difference in knowledge, attitude, practice, and overall KAP [group 1: *t*(29) = 15.73, *p* < 0.01; group 2: *t*(59) = 7.18, *p* < 0.01] in the pre-post-tests in both groups, pointing to the good efficacy of the program. Cohen’s *d* indicates a significant effect size for the KAP of volunteering in both groups. The results emphasize the relevance of mobilizing students for volunteering by enhancing their awareness and practice of volunteering. We recommend adding this training program as part of extra-curricular activities in academic institutions across Oman and similar contexts. Facilitating voluntarism among university students has implications for contributing to Sustainable Development Goals (SDGs) through education for sustainable development efforts envisaged in targets 4.7 and 17.7.

## Introduction

Volunteering is an important skill that helps individuals develop personally and professionally by enhancing health and well-being (Khasanzyanova, 2017). This skill is defined as “a helping action of an individual that is valued by him or her, and yet is not aimed directly at material gain, or mandated or coerced by others” (Van Til, 1988, p. 6). This can be in different forms in terms of organization (formal and informal) and orientation (self or other-oriented) that is determined by human, social, and cultural capitals (Haski-Leventhal, 2014; Yeung et al., 2017; Meijeren et al., 2023). In addition, the act of volunteering is determined by various factors such as age, context, socio-economic status, marital status, social network size, educational level, religiosity, previous volunteering experiences, attitude, motivation and personality, other social participation, situational variables (Smith, 1994; Niebuur et al., 2018; Walker et al., 2022; Van Poppel, 2023; Aslan and Tuncay, 2024a). Further, the perspective on volunteering played a crucial role in its motivation and practice, which could be understood in terms of personal, family, and social environment and society’s perspective (Aslan and Tuncay, 2024b). A qualitative study identified the motivators of volunteering in Turkey’s child welfare as personal experiences and interpersonal aspects such as friends, family, and community perspectives (Aslan and Tuncay, 2023b). Noteworthily, the involvement in volunteering affected the individuals at the bio-psycho-social levels (Aslan and Tuncay, 2023b; Khasanzyanova, 2017).

Volunteering as an act not only benefits the individual in need but also facilitates good physical and mental health, along with promoting subjective, social well-being and life satisfaction among the volunteers (Binder and Freytag, 2013; Yeung et al., 2017; Nichol et al., 2024). A qualitative study evidenced that volunteering provided an enabling environment that allowed better health and well-being to the volunteers by improving their sense of self, community, and connection with others (Turk et al., 2022). Involvement in volunteering activity was associated with well-being and happiness (Lawton et al., 2021). Also, volunteering is associated with a better sense of control, self-esteem, life satisfaction, and physical health, leading to greater well-being (Thoits and Hewitt, 2001). Due to these health benefits, volunteering is considered a social prescription in health care (Tierney et al., 2022).

Individuals’ involvement in volunteering behavior was relatively stable across the life course, with the increase in positive benefits of volunteering over time (Lancee and Radl, 2014; Jiang et al., 2019). Volunteering has a cumulative effect on health and well-being (Yeung et al., 2017), suggesting that early involvement in volunteering activities and practice as a youth could create a greater positive impact throughout life. Involvement in volunteering at a young age facilitated greater social support, stronger values, the possibility to be involved in future volunteering activities, and confidence and a positive image of volunteering compared to their counterparts who did not volunteer (Dávila de León et al., 2020). In addition, youth volunteers are expected to be relationship-oriented, which adds more benefits to volunteering service, especially when the clients are at-risk youths (Haski-Leventhal et al., 2008), providing a mutual benefit for the volunteer and the client.

Although the benefits of volunteering are established, not all are involved in volunteering practices. However, with global concerns such as the rising burden of chronic diseases (Hacker, 2024; World Health Organization [WHO], 2023b), aging population (World Health Organization [WHO], 2024), and climate change (World Health Organization [WHO], 2023a), to name a few, there is a greater need for human resources as volunteers to tackle the problems effectively. In addition, volunteering benefits the community at various levels, including business (Dempsey-Brench and Shantz, 2022), education (Khasanzyanova, 2017; Cerbin-Koczorowska et al., 2022), volunteering for environmental issues and sustainability (Sextus et al., 2024; Wong, 2024) and for leisure activities (Smith et al., 2016; Stebbins, 2023) that promote youth participation at various community activities.

Oman, being one of the developing nations, is not an exception from these global concerns and faces the double burden of an increasing older adult population (Islam, 2020) and chronic diseases (Al Hinai et al., 2020) along with environmental issues and various other crises. In addition, Omani youth’s poor involvement in volunteering services is attributed to various psycho-social factors, including lack of training and guidance (Al Hinai, 2017), suggesting a greater need for youth programs that protect youths from intellectual and behavioral deviation (Alhaj et al., 2022). Although the benefits of volunteerism were evident, it was determined by various micro and macro-level factors that challenged and acted as a barrier to the development and sustainability of voluntary practices (Aslan and Tuncay, 2023a). This points to the need for more involvement in volunteering activities and to raise awareness. With this foundation, the current study aimed to develop and test the efficacy of a training program on the knowledge, attitude, and practice (KAP) of volunteers among university students in Oman. Noteworthily, volunteerism can facilitate promoting Sustainable Development Goals (Ellis Paine et al., 2020), and volunteers play a crucial role in the sustainability of various community-based projects, aiding SDGs (Gowri and Abdul Azeez, 2025; Predețeanu-Dragne et al., 2019; Abdul Azeez and Anbuselvi, 2019). Volunteerism has the potential to facilitate the promotion of the SDGs targets 4.7 and 17.17, which focus on education for sustainable development and promoting effective partnership in society. A detailed description of the intervention program is discussed in the next section.

## Methods

### Research design

We adopted a quasi-experimental design to understand if specialized training programs on volunteering made any difference in university students’ knowledge, attitude, and practice (KAP). Specifically, we used a one-group pre-test-post-test design (with two independent groups at different time points: group 1 and group 2) to evaluate the impact of a training program on the KAP of volunteering among students. This design would be appropriate and suitable for deciphering the role of training programs and evaluating learning as adopted in the study conducted by Araujo et al. (2019). The training module and tool of KAP of volunteering (pre-post-test measure) were developed by the research team through an extensive review of the literature and experts’ validation. The training program was independently delivered to two groups of students (30 students in group 1 and 60 students in group 2) during the fall and spring semesters of 2023 at Sultan Qaboos University.

### Tools

The KAP of volunteering was assessed pre- and post-training using the tool specifically developed for this population and purpose. The research team invited a few experts from the field who were experts in voluntarism and psychometric test development. The experts evaluated the KAP tool of volunteering to check its content and face validity. After the experts’ evaluation and opinions, the final KAP of the volunteering tool had 33 statements (11 each for Knowledge, Attitude and Practice) with a response pattern of 1 to 3 (1 = *limited*; 3 = *strong*). A higher score on each of these components suggests higher knowledge, positive attitude, and good practice of volunteering, while the overall score points to a higher KAP of volunteering. The measure shows good internal consistency for both groups, with the full measure Cronbach’s Alpha of 0.89 and 0.93 in group 1 and group 2, respectively. Further, for each component (KAP), the Cronbach alpha ranged between 0.64 and 0.87 in group 1 and 0.83 and 0.91 in group 2.

### Participants and recruitment

The students from the College of Arts and Social Sciences at Sultan Qaboos University, with no earlier training or academic volunteering background, were randomly selected using a computer-assisted lottery method from a population size of N = 350. The first group consisted of 30 students, and the program was implemented during the fall of 2023. The second group consisted of 60 students, and the program was implemented during spring 2023. Students who had earlier exposure to volunteering through fieldwork or academic background were excluded from the study, as only students with no prior knowledge and experience of volunteering were included. We have included only students without prior volunteering experience because students with volunteering experience will have better knowledge, attitude, and practice in volunteering. Hence, including them could inflate the results. Further, to delimit the scope of the study, we have chosen participants from the arts and social sciences disciplines.

### Intervention

**Figure 1:**
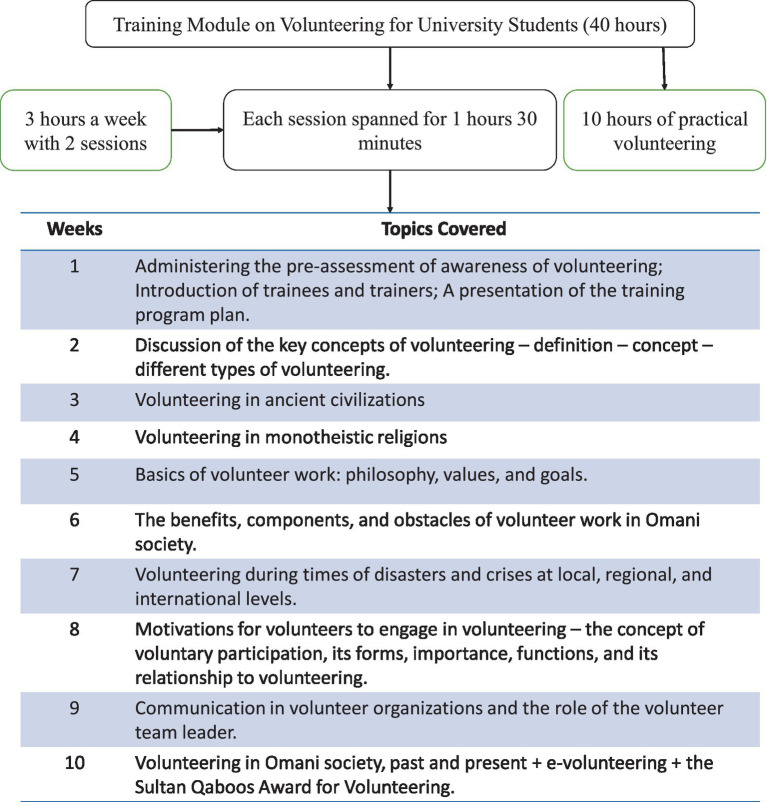
Summary of the training program on volunteering.

A 40-h specialized training on volunteering was given to two different groups of students, with 3 h a week. Further, the 3 h a week were broken down into two sessions of 1 h and 30 min. The training spanned for 10 weeks and covered various aspects of volunteering, including history, components of volunteering, obstacles and needs in the current scenario, and its role in monotheistic religions (refer to Figure 1 for the topics covered in 10 weeks of the program). The participants were also provided a supervised 10-hour practical volunteering opportunity based on their area of interest (health, environment, sensitization). The training modules draw features from United Nations Volunteers (UNV), such as culture-sensitivity, local and community dynamics, crisis management, e-volunteering (UN Volunteering, 2018), and exposure to writing volunteer project proposals and presentations. In addition, parallel to UNV advocacies, this training program promotes mobilizing and increasing the number of volunteers through enhancing the KAP of volunteering among students (United Nations Volunteers [UNV], 2013). The major objectives of the training program were to (1) equip students with knowledge, positive attitudes, and skills related to volunteering and prepare them for voluntary participation; (2) to introduce students to key concepts related to volunteering, such as volunteering, participation, etc., (3) familiarizing trainees with the fundamentals of social volunteer work (philosophy-goal-benefits-components-obstacles, etc.), (4) to enhance the students’ emotional awareness of the importance of social volunteer work in social life; (5) to develop some practical volunteering participation skills among students. A group of qualified professors (who had formal volunteering training) with professional social work backgrounds carried out the training.

### Data analysis

We employed descriptive statistics to understand the characteristics of the sample, and a paired t-test was used to identify the efficacy of a training program on the KAP of volunteering among students. In addition, we calculated Cohen’s d to evaluate the effect size between the pre-post-test of the groups. All the analyses were conducted using SPSS 25.

### Ethical considerations

The study followed all the required protocols per Helsinki’s declaration, and the participants were well informed about their right to withdraw from the study without any negative outcomes. Written informed consent was taken from all the participants before the initiation of the pre-test. Necessary ethical approval was obtained for the conduct of this research.

## Results

The present study tried to understand the role of the volunteer training program (40 h) on the KAP of volunteering among two groups (Group 1, *n* = 30; Group 2, *n* = 60) of students at different points of time. Table 1 presents the descriptive characteristics of the two groups. The mean age of students in group 1 was 21.50 (SD = 1.48), and 11 were male, while 19 were female. In terms of the department, 11 and 9 students belonged to languages and information and communication, respectively, while 7 students belonged to a social sciences background. Group 2’s mean age was 21.32 (1.40), with 25 males and 35 females. Further, 27 and 22 students were in the languages and social sciences, respectively.

**Table 1:** Descriptive statistics of the sample of Group 1 and Group 2.

Variables	Mean/frequency	*SD/%
Group 1 (*n* = 30)		
Age	21.50	1.48
Gender		
Male	11	36.7
Female	19	63.3
Departments		
Arts and Music	3	10
Information and Communication	9	30
Languages	11	36.7
Social Sciences	7	23.3
Group 2 (*n* = 60)		
Age	21.32	1.40
Gender		
Male	25	41.7
Female	35	58.3
Departments		
Arts and Music	1	1.6
Information and Communication	10	16.7
Languages	27	45
Social Sciences	22	36.7

*SD = Standard Deviation; % Percentage.

The summary of the paired t-test of group 1 is presented in Table 2. It is evident from the results that there existed a significant difference between the pre and post-training scores of knowledge [*t*(29) = 20.28, *p* < 0.01], attitude [*t*(29) = 11.24, *p* < 0.01] and practice [*t*(29) = 9.96, *p* < 0.01] and overall KAP [*t*(29) = 15.73, *p* < 0.01]. In addition, the results of Cohen’s *d* suggested a significantly larger effect size on knowledge (*d* = 3.75), attitude (*d* = 2.07), practice (*d* = 1.83), and overall KAP (*d* = 2.88) on volunteering.

**Table 2:** The summary of the paired *t*-test of group 1 considering the awareness of volunteering before and after the training.

Pairs	Mean	SD	Difference			*t*	Cohen’s *d*
			Mean	SD	SE		
Pre-knowledge	17.73	3.16	−14.16	3.82	0.69	20.28**	3.75
Post-knowledge	31.90	2.49					
Pre-attitude	20.90	4.77	−10.33	5.03	0.91	11.24**	2.07
Post-attitude	31.23	2.83					
Pre-practice	16.73	4.66	−8.03	4.41	0.80	9.96**	1.83
Post-practice	24.77	4.64					
Pre-KAP	55.37	10.11	−32.53	11.32	2.06	15.73**	2.88
Post-KAP	87.90	7.69					

Degrees of freedom (df) = 29; **p < 0.01.

Table 3 presents the summary of the paired t-test results of group 2. The results suggest a significant difference between pre-and post-training scores of overall KAP [*t*(59) = 7.18, *p* < 0.01] and its components as knowledge [*t*(59) = 8.77, *p* < 0.01], attitude [*t*(59) = 3.37, *p* < 0.01] and practice [*t*(59) = 4.69, *p* < 0.01]. Further, the effect size test with Cohen’s *d* pointed at the larger effect size for knowledge (*d* = 1.14) and overall KAP (*d* = 0.93), while attitude (*d* = 0.44) and practice (*d* = 0.61) had a moderate effect size for the tests, respectively.

**Table 3:** The summary of the paired *t*-test of group 2 considering the awareness of volunteering before and after the training.

Pairs	Mean	SD	Difference			*t*	Cohen’s *d*
			Mean	SD	SE		
Pre-knowledge	23.23	4.65	−7.35	6.48	0.83	8.77**	1.14
Post-knowledge	30.58	3.74					
Pre-attitude	28.55	3.77	−2.13	4.90	0.63	3.37**	0.44
Post-attitude	30.68	3.41					
Pre-practice	20.17	5.72	−4.48	7.39	0.95	4.69**	0.61
Post-practice	24.65	4.70					
Pre-KAP	71.95	11.60	−13.96	15.05	1.94	7.18**	0.93
Post-KAP	85.92	9.17					

Degrees of freedom (df) = 59; **p < 0.01.

## Discussion

The present study tried to develop a training program to improve the KAP of volunteering and to test its efficacy among university students in Oman. The results from the paired *t*-test suggested a significant difference between the pre-and post-tests in terms of knowledge, attitude, and practice of volunteering and overall KAP. Further, the significant increase in KAP of volunteering after training in both groups at different semesters pointed to the efficacy of the training program. This study is one of its kind to develop and test a tailored training program on volunteering for Omani students. To our knowledge, no earlier works have developed and tested a general training program in this context. However, a systematic review suggested that skill-based volunteering programs can promote the involvement of employees in volunteering activities to support non-profit organizations (NGOs) (Dempsey-Brench and Shantz, 2022). In addition, a study conducted in Russia identified a substantial gap in the training of volunteers for social services and proposed a need for vocational training programs and certification for volunteers in social services, as they require specialized skills, knowledge, and abilities to volunteer in this field (Ogorodnikova et al., 2021).

In the context of Oman, volunteering is considered to be a key value to be promoted among students. Educational institutions are expected to promote the volunteering work culture by suggesting an adopted physical education course for promoting volunteering work with Omani learners with disability (Al-Hadabi et al., 2024). In this line, the results of this study provided a training program on volunteering that promoted KAP of volunteering among students from different educational backgrounds. Further, the decrease in the proportion of students’ involvement in volunteering (Normah and Lukman, 2020) necessitates a training program specially tailored to the local cultural context to promote awareness of volunteering and involvement. As evidenced by the literature, involvement in volunteering not only aids community development but also benefits the individual at different levels, as mentioned before, and reduces the risk of mortality (O'Reilly et al., 2017). In addition, volunteering is considered an equalizer as involvement in it enhances the health and well-being of individuals with low wealth compared to their counterparts (Kim and Halvorsen, 2021). However, a study considering role of NGOs in volunteering evidenced the link between social returns and volunteering activity, the benefits provided to volunteers played role and identified Dhofar governorate to have higher involvement in volunteering practices compared to other governances pointing at the need for policy regulation to safeguard the rights of volunteers and to promote volunteerism among youth in Oman (Al-Ani et al., 2017).

Considering the individual and societal benefits of volunteering, the results of the present study provide a training program for the Omani student population and evidence of the efficacy of this program on volunteering awareness and practice. Voluntarism has explicitly reciprocal benefits for the emerging adults and society. It helps emerging adults to use their time productively and contribute significantly to the global agendas, such as the SDGs. Specifically, involvement in voluntary efforts can enhance the knowledge and skills necessary to promote better attitudes and practices in sustainable lifestyles, gender equality, human rights, peace and non-violence to promote effective public-private and civil society partnership, as specified in SDGs 4.7 and 17.17. This study indicates that the very idea of education for sustainable development could effectively achieve these targets through university education efforts.

### Limitations

This study holds its strength in terms of developing a training program aligned with certain features of UNV and tries to promote them among Omani students. As no work is without limitations, this study also holds certain limitations. First, the training module is tailored explicitly for Oman society (and similar contexts) and cannot be replicated in other contexts without modification. Second, this training module covers general volunteering areas and is not developed for one specific field of volunteering. Third, this study did not test the prolonged efficacy of this training program on volunteering among students, as an immediate pre-post test design was utilized. The inherent limitations of quasi-experimental design apply to this study, as there was no control group. Fourth, the study does not consider the participants’ personality and motivational factors that could possibly influence the results. The differences in effect size of some aspects of the KAP of the two intervention groups could be attributed to the uncontrolled extraneous variables, such as the point of the academic period, in which the pre-test, intervention and post-tests were done. Also, the students’ features, including the years of study at the university, might have been influenced. Students in the program’s initial years might have a lower KAP of volunteering than their counterparts due to the lack of exposure to volunteering activities. However, this needs to be empirically examined in future studies. To overcome these limitations, future studies should consider having a control group and opt for a study design that controls potential extraneous variables, including students’ specific features. In addition, future studies should also consider recording the long-term follow-up of the KAP of the volunteering intervention, which could support the sustainability of the effects.

## Conclusion

The study aimed to develop and test the efficacy of a training program on KAP of volunteering among students in Oman. The results suggested a significant improvement in KAP of volunteering among students in the post-training phase compared to the pre-test, suggesting the efficacy of the program. In the current scenario, the result of this study plays a pivotal role in mobilizing students for volunteering by enhancing the awareness and practice of volunteering. We recommend adding this training program on volunteering as part of extra-curricular activities in academic institutions across Oman and similar contexts. This could strengthen the community and facilitate the development of soft skills among the students. Further, future studies could focus on developing specific training programs for different fields of volunteering. In addition, a model can be created to identify students’ skills that provide opportunities for skills-based volunteering. Facilitating voluntarism among university students has implications for contributing to Sustainable Development Goals (SDGs) through education for sustainable development efforts envisaged in targets 4.7 and 17.7. The evidence from the present study indicates that university education efforts can be instrumental in sensitizing students about critical social issues and enhancing their skills, which may contribute to achieving a sustainable society and the SDGs.

## Data Availability

The raw data supporting the conclusions of this article will be made available by the authors, without undue reservation.
